# Design and Research of a New Cold Storage: The Phase-Temperature Storage (PTS) to Reduce Evaporator Frosting

**DOI:** 10.3390/foods14091592

**Published:** 2025-04-30

**Authors:** Lihua Duan, Yanli Zheng, Yunbin Jiang, Wenhan Li, Limei Li, Bin Liu, Bin Li, Xihong Li

**Affiliations:** 1 College of Mechanical Engineering, Tianjin University of Science and Technology, Tianjin 300457, China; 2 Institute of Agricultural Products Preservation and Processing Science and Technology, Tianjin Academy of Agricultural Sciences, Tianjin 300384, China; zhengyanli3344@163.com; 3 Chestnut Research Center, Hebei Normal University of Science and Technology, Qinhuangdao 066004, China; 4 State Key Laboratory of Food Nutrition and Safety, Tianjin University of Science and Technology, Tianjin 300457, China; 5 International Centre in Fundamental and Engineering Thermophysics, Tianjin University of Commerce, Tianjin 300134, China; lbtjcu@tjcu.edu.cn (B.L.);

**Keywords:** phase-temperature storage, conventional cold storage, energy saving, precision temperature control, sub-storehouse, mother chamber, evaporator, frosting

## Abstract

This paper introduces a novel cold storage: phase-temperature storage, which is characterized by its distinctive coupling jacket structure that connects the sub-storehouse units to the main storehouse. This innovative design facilitates heat transfer while effectively inhibiting mass transfer. Experimental results indicate that polyethylene film, with a thermal conductivity of 0.42 W/m·K, is a more suitable material for constructing sub-storehouses. Enhancing the surface area of the sub-storehouse and increasing convective wind speed are identified as key factors for improving convective heat transfer within the sub-storehouse. Moreover, the optimized design ensures a more uniform temperature distribution inside the sub-storehouse. In contrast to conventional cold storage, the defrosting unit in phase-temperature storage consumes only 5.72 units of energy under equivalent conditions, compared to 154.02 units for conventional cold storage. This demonstrates that the energy expenditure during the defrosting process of phase temperature storage is less than 4% of that required by conventional cold storage, achieving an energy savings rate exceeding 96%. Under identical circumstances, conventional cold storage consumes a total of 36.359 units of electrical energy for defrosting, with 34.231 units being released as defrosting waste heat into the cold storage environment, resulting in a loss rate of approximately 94.13%. Based on apple preservation experiments, phase-temperature storage exhibited significantly superior performance compared to conventional cold storage in terms of apple respiratory peak, weight loss rate, hardness, and TSS content, with respective values of 17.05 CO_2_ mg·kg^−1^·h^−1^, 2.89%, 9.29 N, and 16.3%. In contrast, the conventional cold storage group recorded values of 18.15 CO_2_ mg·kg^−1^·h^−1^, 5.16%, 8.42 N, and 14.9%. These results highlight the exceptional freshness-retention capabilities of phase-temperature storage, underscoring its considerable potential for application in storage systems.

## 1. Introduction

Global grain production totals approximately 5.479 billion tons, with post-harvest losses estimated at around 14%, equivalent to roughly 767 million tons. Fruits and vegetables have a global production of 2.035 billion tons, of which developing countries account for 1.821 billion tons (89.5% of the worldwide total). Annually, losses in fruits and vegetables are estimated to be approximately 30%, amounting to roughly 610.5 million tons per year. These losses in fruits, vegetables, and grains could potentially provide sufficient nutrition for approximately 3 billion people, representing about 40% of the global population. Therefore, the scale of these losses is significant and warrants attention [1]. Maria [2] and Zou [3] highlighted that temperature is a pivotal factor influencing the composition and concentration of aroma compounds, as well as impacting respiration, transpiration, oxidation, and antioxidant properties of fruits and vegetables post-harvest. This variable can accelerate senescence and cellular degradation processes. Chen [4] and Deng [5] examined the effects of temperature fluctuations and found that minimizing these variations significantly improves preservation efficacy. Chen [6] further demonstrated that precise temperature control markedly slows down the deterioration of apple firmness. Xin [7] observed that temperature instability negatively affects the brightness, color, and texture of sweet cherries. Such instability also leads to elevated expression levels of paB-Gal1 and paB-Gal3 genes and increased dB-Gal activity during subsequent constant-temperature storage. These findings emphasize the necessity of maintaining stable temperatures to ensure the quality of fruits and vegetables. Effective regulation of storage temperature is essential, as it directly contributes to preserving produce quality and ensuring food safety [8]. To address this challenge, Makule [1] and Sonawane [9] improved the thermal insulation and energy efficiency of cold storage systems through optimized structural designs. Research [10,11,12,13,14,15] indicates that upgrading insulating materials not only conserves energy but also ensures consistent temperature maintenance. Moreover, studies [16,17,18] suggest that incorporating materials with low thermal conductivity, such as polyurethane foam or glass wool, into the exterior of storage facilities provides enhanced thermal protection. Nevertheless, from the perspective of preventing frost accumulation on evaporators, only a limited number of researchers have successfully achieved simultaneous optimization of temperature control, energy efficiency, and preservation quality in cold storage systems. The primary causes of imprecise temperature regulation and increased energy consumption in cold storage facilities [19,20] are strongly linked to evaporator frosting [21]. This phenomenon arises due to elevated moisture levels within the storage environment. Moreover, frost formation obstructs airflow, leading to reduced operational efficiency [22,23] and higher refrigeration energy demands. However, maintaining the freshness of fruits and vegetables stored in the warehouse [24] necessitates preserving a high-humidity environment. As a result, mitigating evaporator frosting under these conditions presents a significant challenge. In this study, an innovative solution for cold storage, referred to as “phase-temperature storage”, is proposed to address this issue.

The concept of phase-temperature storage refers to a refrigeration system that integrates a jacket layer with a primary storage unit. Although similar jacket layers have been in use for a long time [25], their application in cold-storage systems is still relatively rare. The main storehouse unit is constructed using 100 mm-thick double-sided color steel polyurethane insulation panels, creating a thermally insulated and air-tight enclosure. The key innovation of the phase-temperature storage system lies in embedding the main storehouse unit within a sub-storehouse unit, with the evaporator located between the jacket layers of the main storehouse. While the material of the sub-storehouse unit does not require thermal insulation, it must ensure airtightness. The term “non-insulated” indicates that the cooling effect from the jacket layer of the main storehouse can be transferred into the sub-storehouse unit via its walls, thereby indirectly cooling its contents. Moreover, the respiratory heat generated by fruits and vegetables in the sub-storehouse can also be transmitted to the jacket layer of the main storehouse through the walls of the sub-storehouse. The term “air-tight” denotes the prevention of moisture transfer from the sub-storehouse to the outer jacket layer, a property primarily determined by the material characteristics of the sub-warehouse. This not only sustains a low-temperature, high-humidity environment suitable for fruit and vegetable storage within the sub-storehouse but also ensures that the evaporator operates under low-humidity conditions, thereby minimizing frost accumulation or achieving frost-free performance. As a result, this improves the temperature control accuracy of the cold storage system while reducing energy consumption during the storage process. Identifying suitable sub-storehouse materials and their associated parameters has become a key focus of our research. Moreover, the precise structure of the phase-temperature storage will be refined through experimental validation, its energy-saving potential will be quantified, and the freshness-preserving effectiveness of the phase-temperature storage will be comprehensively evaluated.

## 2. Materials and Methods

### 2.1. Materials

#### 2.1.1. Experimental Materials

The apples used in this experiment, named Aksu Sugar Heart, were obtained from the Hongqipo Group based in Aksu, Xinjiang, China (200 kg). Meanwhile, the polyethylene film, aluminum foil, and iron plates were purchased from the Jinyuanbao Wholesale Market located in the Binhai New Area of Tianjin, China. The various samples, each measuring exactly 100 m in length, have specific thickness dimensions of 0.2 mm, 0.4 mm, and 0.8 mm, respectively.

#### 2.1.2. Experimental Instruments and Equipment

The experimental phase utilized various facilities and instruments provided by different manufacturers. Temperature-controlled storage and conventional cold storage facilities were supplied by Tianjin, China Jiesheng Donghui Fresh-keeping Technology Co., Ltd. An intelligent temperature controller (model TC-05B) manufactured by Guangzhou Xifa Electronics Co., Ltd. (Guangzhou, China) was employed for precise temperature regulation. The aluminum alloy heater used in the study was the DJR-500S model provided by Yukai Electric Power Technology Co., Ltd. (Leqing, China). A hot-wire anemometer (model QDF-6) from Kaixing Demo Instrument Equipment Co., Ltd. (Beijing, China) was utilized to measure airflow characteristics. The three-phase four-wire meter adopted was the DTS606 model manufactured by Delisi Group Instrument Co., Ltd. (Wenzhou, China), which was applied for power-consumption monitoring. For power monitoring, the P08S model from Ningbo High-tech Zone Xincheng Electronics Co., Ltd. (Ningbo, China) was also employed. The fruit and vegetable respiration tester used was the GXH-3051H model supplied by Shijiazhuang Shiya Technology Co., Ltd. (Shijiazhuang, China). A digital handheld refractometer (model PAL-3) from Zhuogang Instrument Technology Co., Ltd. (Shanghai, China) was utilized for measuring sugar content. The digital hardness tester employed was the GY-4 model from Shenzhen Huafeng Technology Co., Ltd. (Shenzhen, China). Additionally, a digital probe thermometer (Model 11000) from DELTATRAK (Pleasanton, CA, USA) and a high-precision multipoint thermometer (Model F59) from Fluke Test Instruments (Shanghai, China) were used for temperature measurements.

### 2.2. Methods

#### 2.2.1. Determination of the Sub-Storehouse Material for Phase-Temperature Storage

As discussed earlier, the main feature of the sub-storehouse is its lack of insulation while maintaining air-tightness. In other words, the sub-storehouse is designed to transfer heat without transferring mass. Therefore, when determining the material for the sub-storehouse, its heat transfer properties need to be thoroughly understood [26].

Heat Transfer Calculation for sub-storehouse:

Q = Q2 − Q1(1)

Q1: Q1 represents the initial heat value in kJ. Q2: Q2 represents the final heat value in kJ. Q: Q represents the heat transferred in the experiment, in kJ.

**Figure 1:**
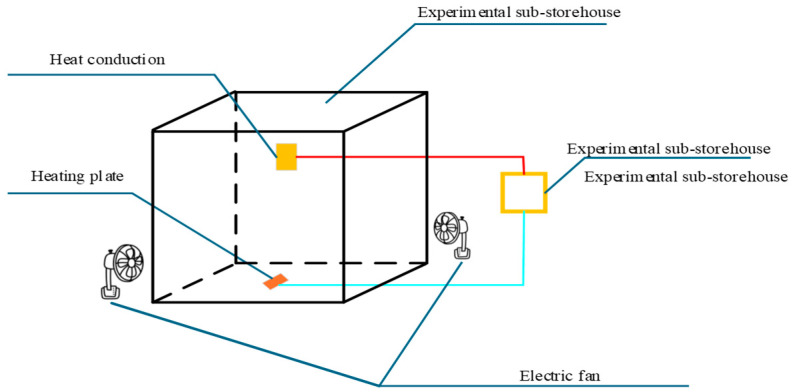
Sub-storehouse Heat Transfer Experiment Device.

The initial experimental parameters are set as follows: sub-storehouse wall thickness, thermal conductivity of sub-storehouse material, convective wind velocity outside the sub-storehouse (provided by fans placed at opposite corners of the sub-storehouse and main storehouse), and heat transfer area of the sub-storehouse. The set values are: 0.2 mm, 0.42 W/(m·K), 3 m/s, and 5 m^2^. The device for the sub-storehouse heat transfer experiment is shown in Figure 1. A cubic sub-storehouse with a volume of 1 m^3^ is designed. Since the bottom of the sub-storehouse does not contribute to the heat transfer area, it is insulated with sufficient insulating materials. An aluminum alloy heating plate is placed inside the sub-storehouse to simulate the respiration heat of fruits and vegetables through heating. The sub-storehouse is then placed into a conventional cold storage with the temperature pre-adjusted to 0 °C. When the temperature inside the cold storage reaches 3 °C, the intelligent temperature controller of the cold storage is activated. When the temperature reaches 5 °C, the intelligent temperature controller turns off the heating plate inside the sub-storehouse. This process is repeated for 24 h. The energy consumption of the heater is recorded, observed, and compared under various conditions, including different wall thicknesses, materials, convective wind velocity outside the sub-storehouse, and heat transfer areas. Since the set temperature of the cold storage is 0 °C, a higher energy consumption of the sub-storehouse indicates better heat transfer performance of the sub-storehouse wall. In other words, the higher the energy consumption of the heating plate, the better the heat transfer performance of the sub-storehouse. Therefore, the energy consumption of the heating plate can be used as a direct measure of the heat transfer performance of the sub-storehouse wall. The experimental approach involves changing the thickness, thermal conductivity, convection wind speed, and heat transfer area of the sub-storehouse wall under the same temperature gradient and time conditions. By comparing the heat transfer of the sub-storehouse wall under these varying conditions, the best material selection for the sub-storehouse wall can be determined.

#### 2.2.2. Simulation of the Temperature Field Within the Phase Temperature Storage and Optimization of the Sub-Storehouse Structure

As previously discussed, temperature has been identified as a critical determinant of stored product quality [27,28]. However, evidence suggests that temperature fluctuations in most conventional cold storage units are significant, with uneven distribution observed even within the same plane [29]. This raises the question of what the temperature distribution is like inside the phase temperature storage. To investigate this, a simulation was conducted.

**Figure 2:**
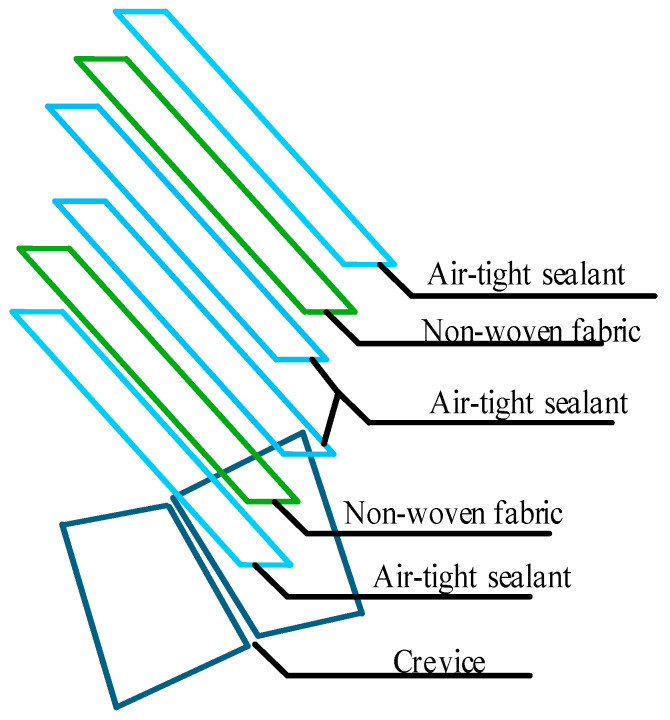
Air-tight Sealing of Sub-storehouse Seams.

**Figure 3:**
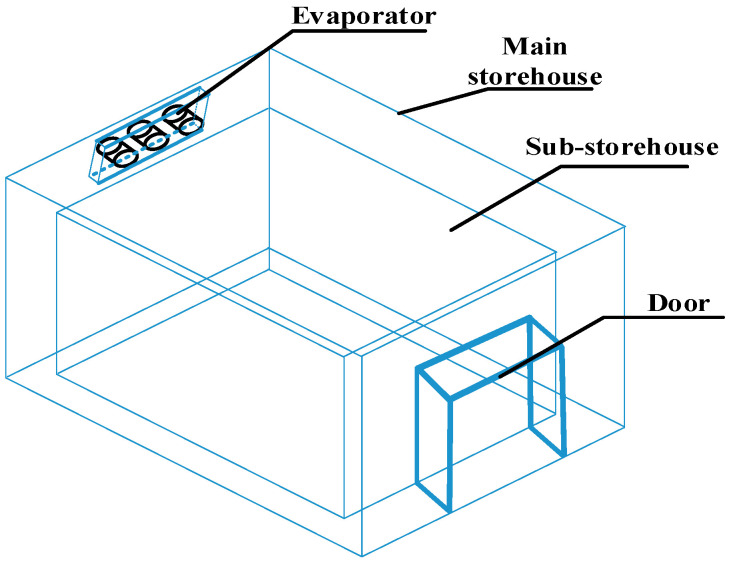
Sub-storehouse Model.

Since an evaporator will be installed in the mother chamber and fresh-keeping experiments will be conducted in the sub-storehouse, the dimensions of the phase-temperature storage mother chamber in this study were set at 9.2 m × 7.2 m × 6 m (L × W × H), while the sub-storehouse dimensions were 8 m × 6 m × 5 m (L × W × H). The gap between the jacket layers of the mother and sub-storehouse was established at 0.6 m, determined based on a comprehensive consideration of fluid mechanics principles and practical factors, with a 1 m gap at the top. To ensure air-tightness, the seams of the sub-storehouse were sealed using a four-layer combination of adhesive and fabric (adhesive + fabric + adhesive + adhesive + fabric + adhesive), as illustrated in Figure 2. The evaporator was positioned in the top jacket layer, with the return air vent located 0.4 m from the mother chamber wall. The evaporator dimensions were 3 m × 0.4 m × 0.8 m (L × W × H), with an air outlet diameter of 0.5 m. It was installed on the ceiling, 0.1 m below the top of the cold storage. Figure 3 presents the physical model of the phase-temperature storage.

### 2.3. Mathematical Model

Within the jacket layer, the forced convection generated by the evaporator was considered to be a high Reynolds number turbulent flow. Consequently, the standard k−ε model was selected, and the mass conservation, energy conservation, and momentum conservation equations were coupled. The fluid flow and heat/mass transfer processes inside the cold storage were simulated using COMSOL Multiphysics^®^6.3, a software based on the finite-element method (FEM) for Computational Fluid Dynamics (CFD). The specific steps involved in this process are described as follows.

1. Standard k−ε model

In the standard k−ε model, k represents the turbulent kinetic energy and ε represents the dissipation rate of turbulent kinetic energy. These two quantities are the primary unknowns, and their relationship is provided by:

(2) $$\mu_{t}=\rho C_{\mu }\frac{k^{2}}{\varepsilon }$$

The corresponding transport equations are expressed as:

(3) $$\frac{\partial \left(\rho k\right)}{\partial t}+\frac{\partial \left(\rho k\mu_{i}\right)}{\partial x_{i}}=\frac{\partial }{\partial x_{j}}\left[\left(\mu +\frac{\mu_{t}}{\sigma_{k}}\right)\frac{\partial k}{\partial x_{i}}\right]+G_{k}+G_{b}-\rho \varepsilon -Y_{M}+S_{K}$$

(4) $$\frac{\partial \left(\rho \varepsilon \right)}{\partial t}+\frac{\partial \left(\rho \varepsilon \mu_{i}\right)}{\partial x_{i}}=\frac{\partial }{\partial x_{j}}\left[\left(\mu +\frac{\mu_{t}}{\sigma_{\varepsilon }}\right)\frac{\partial \varepsilon }{\partial x_{i}}\right]+C_{1}\frac{\varepsilon }{k}\left(G_{k}+C_{3}G_{b}\right)-C_{2}\rho \frac{\varepsilon^{2}}{k}+S_{\varepsilon }$$

Assuming the fluid within the reservoir is incompressible, then $G_{b}=0$,$Y_{M}=0$, $S_{K}=0$, and $S_{\varepsilon }=0$ (where, $G_{b}$ is the additional term for turbulence kinetic energy due to buoyancy, $Y_{M}$ is caused by pulse expansion in compressible turbulence, and $S_{K}$ and $S_{\varepsilon }$ are custom source terms that are neglected here). Then, the equations become:

(5) $$\frac{\partial \left(\rho k\right)}{\partial t}+\frac{\partial \left(\rho k\mu_{i}\right)}{\partial x_{i}}=\frac{\partial }{\partial x_{j}}\left[\left(\mu +\frac{\mu_{t}}{\sigma_{k}}\right)\frac{\partial k}{\partial x_{i}}\right]+G_{k}-\rho \varepsilon$$

(6) $$\frac{\partial \left(\rho \varepsilon \right)}{\partial t}+\frac{\partial \left(\rho \varepsilon \mu_{i}\right)}{\partial x_{i}}=\frac{\partial }{\partial x_{j}}\left[\left(\mu +\frac{\mu_{t}}{\sigma_{\varepsilon }}\right)\frac{\partial \varepsilon }{\partial x_{i}}\right]+C_{1}\frac{\varepsilon }{k}G_{k}-C_{2}\rho \frac{\varepsilon^{2}}{k}$$

In the equations, $G_{k}$ represents the additional term for turbulent kinetic energy caused by the mean velocity gradient $\sigma_{k}$, $\sigma_{\varepsilon }$ are the Prandtl numbers for turbulent kinetic energy k and dissipation rate ε, respectively, with empirical values taken as 1.0 and 1.3; $C_{1}$, $C_{2}$, $C_{3}$ are the constant terms, with empirical values taken as 1.44, 1.92, and 0.09 [30].

2. Mass Conservation Equation

(7) $$\frac{\partial \rho }{\partial t}+\frac{\partial \left(\rho u\right)}{\partial x}+\frac{\partial \left(\rho \nu \right)}{\partial y}+\frac{\partial \left(\rho w\right)}{\partial z}=0$$

Since it was previously stated that the fluid inside the chamber is incompressible, Equation (7) becomes:

(8) $$\frac{\partial \left(\rho u\right)}{\partial x}+\frac{\partial \left(\rho \nu \right)}{\partial y}+\frac{\partial \left(\rho w\right)}{\partial z}=0$$

3. Momentum Conservation Equations

(9a) $$\frac{\partial \left(\rho u\right)}{\partial t}+div\left(\rho uu\right)=-\frac{\partial p}{\partial x}+\frac{\partial \tau_{xx}}{\partial x}+\frac{\partial \tau_{xy}}{\partial y}+\frac{\partial \tau_{zx}}{\partial z}+F_{x}$$

(9b) $$\frac{\partial \left(\rho v\right)}{\partial t}+div\left(\rho vu\right)=-\frac{\partial p}{\partial x}+\frac{\partial \tau_{xy}}{\partial x}+\frac{\partial \tau_{yy}}{\partial y}+\frac{\partial \tau_{zy}}{\partial z}+F_{y}$$

(9c) $$\frac{\partial \left(\rho w\right)}{\partial t}+div\left(\rho wu\right)=-\frac{\partial p}{\partial x}+\frac{\partial \tau_{xz}}{\partial x}+\frac{\partial \tau_{yz}}{\partial y}+\frac{\partial \tau_{zz}}{\partial z}+F_{z}$$

In the equations: P represents the pressure on the fluid element;

τ represents the viscous force;

$F_{x}$$F_{y}$$F_{z}$ represents the volume forces acting on the fluid element.

Since it was previously stated that the fluid inside the chamber is incompressible, Equation (9) becomes:

(10a) $$\frac{\partial \left(\rho u\right)}{\partial t}+\frac{\partial \left(\rho uu\right)}{\partial x}+\frac{\partial \left(\rho uv\right)}{\partial y}+\frac{\partial \left(\rho uw\right)}{\partial z}=-\frac{\partial p}{\partial x}+\frac{\partial }{\partial x}\left(\mu \frac{\partial u}{\partial x}\right)+\frac{\partial }{\partial y}\left(\mu \frac{\partial u}{\partial y}\right)+\frac{\partial }{\partial z}\left(\mu \frac{\partial u}{\partial z}\right)+S_{u}$$

(10b) $$\frac{\partial \left(\rho v\right)}{\partial t}+\frac{\partial \left(\rho vu\right)}{\partial x}+\frac{\partial \left(\rho vv\right)}{\partial y}+\frac{\partial \left(\rho vw\right)}{\partial z}=-\frac{\partial p}{\partial x}+\frac{\partial }{\partial x}\left(\mu \frac{\partial v}{\partial x}\right)+\frac{\partial }{\partial y}\left(\mu \frac{\partial v}{\partial y}\right)+\frac{\partial }{\partial z}\left(\mu \frac{\partial v}{\partial z}\right)+S_{v}$$

(10c) $$\frac{\partial \left(\rho w\right)}{\partial t}+\frac{\partial \left(\rho wu\right)}{\partial x}+\frac{\partial \left(\rho wv\right)}{\partial y}+\frac{\partial \left(\rho ww\right)}{\partial z}=-\frac{\partial p}{\partial x}+\frac{\partial }{\partial x}\left(\mu \frac{\partial w}{\partial x}\right)+\frac{\partial }{\partial y}\left(\mu \frac{\partial w}{\partial y}\right)+\frac{\partial }{\partial z}\left(\mu \frac{\partial w}{\partial z}\right)+S_{w}$$

In the equations: u, v, and w are the velocity components in the x, y, and z directions;

μ represents the laminar viscosity coefficient; $kg/\left(m\cdot s\right)$

k represents the turbulent kinetic energy; $m^{2}/s^{2}$

ε represents the dissipation rate of turbulent energy; $m^{2}/s^{2}$

P represents the pressure; pa

ρ represents the density; $kg/m^{3}$

4. Energy Conservation Equation

(11) $$\frac{\partial \left(\rho T\right)}{\partial t}+\frac{\partial \left(\rho u_{j}T\right)}{\partial x_{j}}=\frac{\partial }{\partial x_{j}}\left[\left(\frac{K}{C_{p}}+\frac{\mu_{i}}{\sigma_{T}}\right)\frac{\partial T}{\partial x_{j}}\right]+S_{T}$$

In the equations: T represents the absolute temperature; K

K represents the heat transfer coefficient of the fluid; $W/\left(m^{2}\cdot K\right)$

$C_{p}$ represents the specific heat capacity at constant pressure; $J/\left(kg\cdot K\right)$

#### 2.3.1. Boundary Conditions

(1) Inlet Boundary: The evaporator air supply inlet is defined as a velocity inlet. The air supply velocity from the evaporator is set to 10 m/s, while the air supply temperature is set to 0 °C.

(2) Outlet Boundary: The return air inlet is set as an open boundary.

(3) Wall Boundary Conditions: The bottom of the cold storage is set as an adiabatic boundary. The walls of the main storehouse are treated with the second type of thermal boundary condition, with a heat transfer coefficient set to 0.4 W/(m^2^·K) [31], and the ambient temperature is set to 20 °C. No-slip conditions are applied to the walls. The sub-storehouse has a thin wall, and if represented directly by geometric domains, it would result in poor mesh quality. Therefore, the thin layer functionality in COMSOL is used to define it as a thermal thin approximation.

#### 2.3.2. Defrosting Experiment Design

The defrosting unit is essentially an electric heating wire connected to the evaporator, where the electric wire generates heat to achieve defrosting [32]. Two cold storage units using the same defrosting unit are designed. The rated power of the electric heating wire is selected to be 5 KW, with the evaporator coil area being 50 m^2^, and the storage temperature maintained at 0 °C with 90% relative humidity. This serves as the baseline for running the system for 7 days (168 h).

##### Determination of Defrosting Energy Consumption

With all other parameters kept constant, dedicated electricity meters are installed on the electric heating wires of the defrosting units in both cold storage units. The electricity meter readings are measured and recorded every 12 h, with the system running continuously for 7 days (168 h). These data are employed to calculate the actual defrosting electricity consumption of the cold storage units.

##### Determination of Defrosting Heat Recovery

In practice, not all the electrical energy consumed by the electric heating wire is used for defrosting. The majority of this energy is dissipated as residual heat within the cold storage, which increases the refrigeration load and causes unnecessary energy loss. Therefore, the residual defrosting heat must be considered. The melted frost water is collected, and its temperature and weight are measured. The defrosting energy is calculated by subtracting the energy used for actual defrosting from the total energy consumed by the electric heating wire. The formula for calculating this is as follows (unit: kWh).

(12) $$W=W_{1}-(c_{1}m\Delta t_{2}+m\gamma +c_{2}m\Delta t_{2})/3.6\times 10^{3}$$

In the equations: W represents the defrosting residual heat;

$W_{1}$ represents the total energy consumed by the electric heating wire;

$c_{1}$ represents the specific heat capacity of ice, taken as 2.15 kJ/(kg·°C);

m represents the weight of the melted frost water

γ represents the heat of fusion of frost, taken as 334 kJ/kg;

$\Delta t_{1}$ represents the temperature difference between the evaporator and 0 °C, taken as 8 °C;

$c_{2}$ represents the specific heat capacity of water, taken as 4.2 kJ/(kg·°C);

$\Delta t_{2}$ represents the temperature difference between the collected defrosted water and 0 °C, taken as 5 °C.

#### 2.3.3. Simulation Analysis of Goods Stored in Sub-Storehouse

**Figure 4:**
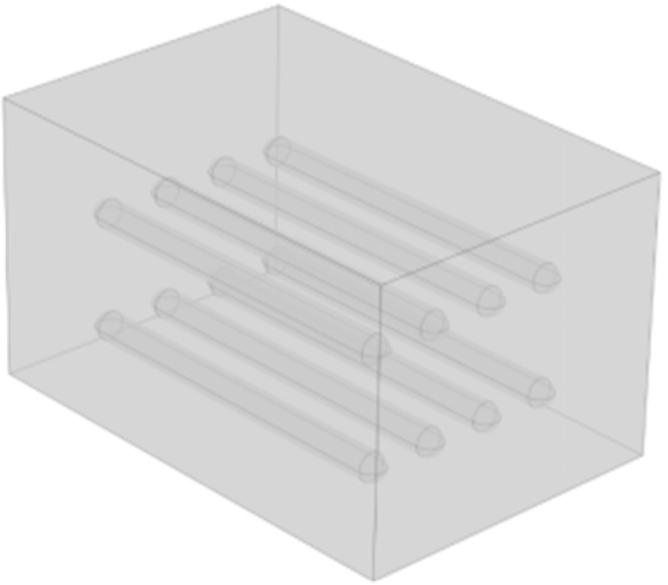
Physical model of a sub-storehouse for storing goods.

The “Conjugate Heat Transfer and Laminar Flow” multiphysics interface in COMSOL Multiphysics was coupled with the surface-to-surface radiation interface to enable precise simulation of airflow and heat transfer within the sub-storehouse. The walls of the sub-storehouse were maintained at a constant temperature, indicating no thermal interaction between the interior and exterior environments. As such, the sub-storehouse walls were defined using Dirichlet boundary conditions (Type I). The internal items were modeled as cylindrical objects with dimensions of 0.5 m in diameter and 6.6 m in height, arranged with a spacing of 2 m between cargo layers. The lowest cargo layer was positioned 1 m above the floor. The sub-storehouse dimensions were specified as 8 m × 6 m × 5 m (length × width × height), as shown in Figure 4.

The freezing point of common fruits and vegetables under normal pressure is shown in Table 1, all of which are below −1 °C. In order to prevent cold damage, the wall temperature is set at −1 °C.

Due to the influence of various factors, the surface emissivity ε of polyethylene film materials typically ranges from 0.8 to 0.95. For a more conservative estimation, we assume ε = 0.8 for subsequent analysis. The respiratory heat generated by different commodities exhibits significant variation. Existing studies consistently indicate that at temperatures below 10 °C, the unit storage respiratory heat for most fruits and vegetables remains below 16 W/m^3^. Therefore, to investigate the impact of respiratory heat on the temperature distribution and velocity field within the sub-storehouse, the internal heat source q of the goods is classified into four levels: 4 W/m^3^, 8 W/m^3^, 12 W/m^3^, and 16 W/m^3^.

#### 2.3.4. Design of the Freshness Preservation Experiment

Following apple harvesting and field heat dissipation, the apples were packed in boxes. Upon arrival at the laboratory the following day, the boxes were immediately opened for pre-cooling, which involved placing them in a 0 °C environment for 24 h. Apples with a smooth appearance, similar size, uniform maturity, and absence of injuries were selected for the experiment. The apples were grouped into sets of 6–7, with each group placed into a PE freshness-preserving bag. Ten bags were placed in both the conventional cold storage and the phase-temperature storage, all positioned centrally within the storage, 10 cm above the ground. The storage temperature was set to 0 °C with a relative humidity of 90%. Subsequently, samples were randomly taken every 20 days, and related indicators were measured to compare the preservation effects of different cold storage types.

##### Measurement of Apple Respiration Intensity

The respiration intensity is measured using the respiration intensity tester selected from Table 2, with units: [unit: CO_2_mg·kg^−1^·h^−1^].

##### Measurement of Apple Weight Loss Rate

The weight loss rate of apples is calculated using the weight method. The calculation method is shown in Formula (13). The unit for weight loss rate is %.

(13) $$\mathrm{Weight}\;\mathrm{loss}\;\mathrm{rate}=\frac{\mathrm{Weight}\;\mathrm{before}\;\mathrm{storage}-\mathrm{Weight}\;\mathrm{after}\;\mathrm{storage}}{\mathrm{Weight}\;\mathrm{before}\;\mathrm{storage}}\times 100\%$$

##### Measurement of Apple Hardness

Five apples were randomly selected. For each apple, three points were uniformly chosen along the equator, and the hardness tester probe was inserted perpendicularly to the apple’s surface at a steady speed. Each apple was measured three times, and the average value was recorded. The unit for hardness is [unit: $\mathrm{kg}\cdot {\mathrm{cm}}^{-2}$].

##### Measurement of Soluble Solid Content (TSS) in Apples

One apple was randomly selected, and a portion of its flesh was cut along the equator, juiced, and filtered. The TSS content of the filtered juice was measured using a refractometer. This procedure was repeated three times, and the average value was recorded. The unit for TSS content is %.

##### Experimental Data Processing

Statistical significance analysis of the experimental data will be conducted using SPSS24.0 software. The velocity and temperature distribution within the cold storage will be studied using CFD simulation software COMSOL Multiphysics. Origin 9.0 software will be used to generate charts for data visualization.

## 3. Results

### 3.1. Determination of Sub-Storehouse Body for Phase-Temperature Storage

**Figure 5:**
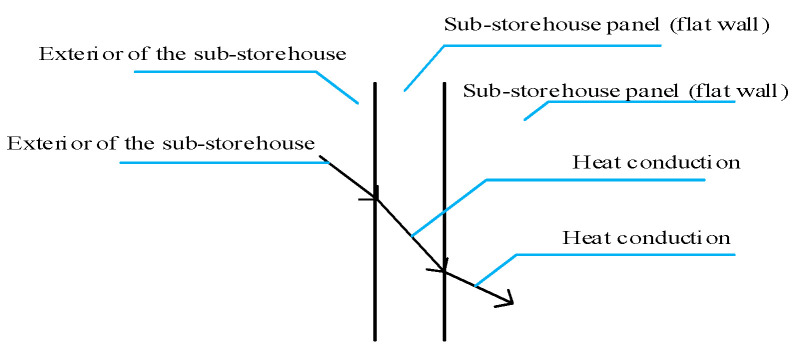
Schematic of Heat Transfer at Sub-storehouse Wall.

It is known that temperature difference serves as potential energy and is transferred through conduction, radiation, and convection in three-phase media (gas, liquid, and solid) [33,34,35]. The heat transfer process in the sub-storehouse is no exception, as shown in Figure 5. This process involves wall heat transfer [36], and since the temperature in the phase temperature storage is very low, the radiation heat can be neglected [37]. Therefore, convection and conduction are identified as the primary factors influencing heat transfer in the sub-storehouse. Convection heat transfer is related to the heat transfer area and airspeed, while conduction depends on the thermal conductivity of the sub-storehouse material and the thickness of the material [36]. Consequently, the heat transfer area, thermal conductivity, thickness of the sub-storehouse material, and external wind speed of the sub-storehouse have been chosen as the main research variables in the heat transfer process.

(a) Selection of Sub-storehouse Body Materials

**Figure 6:**
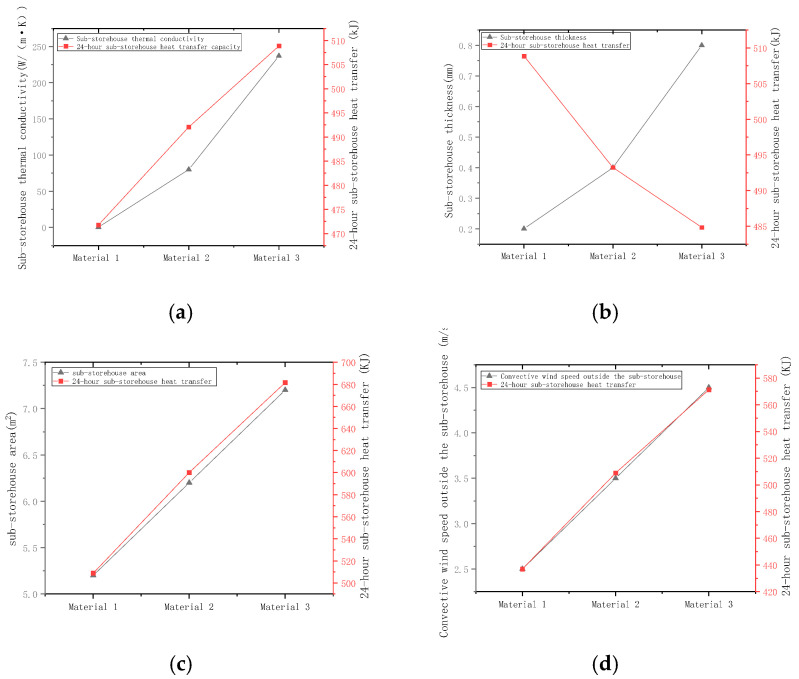
Sub-storehouse Material Parameter Determination. (**a**) Relationship between sub-storehouse heat transfer and thermal conductivity of sub-storehouse material; (**b**) Relationship between sub-storehouse heat transfer and sub-storehouse thickness; (**c**) Relationship between sub-storehouse heat transfer and heat transfer area of sub-storehouse; (**d**) Relationship between sub-storehouse heat transfer and convective wind speed.

Based on preliminary market research, three cost-effective materials—polyethylene film, iron sheet, and aluminum sheet—were chosen for investigation, with a known thermal conductivity of 0.42 W/(m·k), 80 W/(m·k), and 237 W/(m·k), respectively. Under the initial conditions of sub-storehouse wall thickness = 0.2 mm, external convection velocity = 3 m/s, and sub-storehouse heat transfer area = 5 m^2^, the heat transfer rates of the sub-storehouse over 24 h were measured to be 471.77 kJ, 492.05 kJ, and 508.88 kJ, respectively. The relationship between heat transfer rate and thermal conductivity is depicted in Figure 6a.

(b) Determination of Sub-storehouse Wall Thickness

Generally, increasing the sub-storehouse’s wall thickness enhances its mechanical strength and stability. However, this also leads to increased costs. A balance must be achieved between these factors. The question arises whether this characteristic can be used to determine the thickness of the reservoir wall. To investigate this, a series of experiments was conducted. Firstly, other parameters were maintained as their initial conditions (thermal conductivity of the sub-storehouse wall material: 0.42 W/(m·k); convective wind speed outside the sub-storehouse: 3 m/s; heat transfer area of the sub-storehouse wall: 5 m². Then, the heat transfer quantities of sub-storehouses with wall thicknesses of 0.2 mm, 0.4 mm, and 0.8 mm were measured and recorded over a 24 h period. The values obtained were 508.85 kJ, 493.25 kJ, and 484.84 kJ, respectively. The relationship between heat transfer rate and wall thickness is illustrated in Figure 6b.

(c) Determination of sub-storehouse Heat Transfer Area

**Figure 7:**
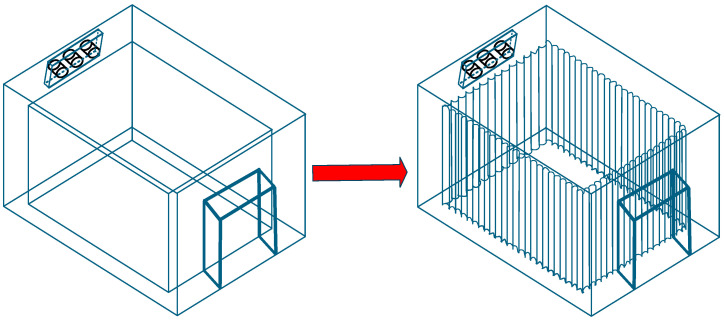
Sub-storehouse Wall Surface Model: Flat to Corrugated Surface.

As previously mentioned, the heat transfer of the sub-storehouse primarily occurs through flat wall heat transfer, which is proportional to the heat transfer area [38,39]. Therefore, understanding the relationship between the chamber’s heat transfer area and its heat transfer performance is crucial. To increase the heat transfer area without altering the sub-storehouse’s volume, the chamber’s walls were modified from flat to corrugated surfaces, as shown in Figure 7. By adjusting the corrugation curve, different surface areas were obtained. The measured heat transfer rates over 24 h for areas of 5.2 m^2^, 6.2 m^2^, and 7.23 m^2^ were 508.90 kJ, 600.10 kJ, and 681.61 kJ, respectively. The relationship between heat transfer rate and surface area is presented in Figure 6c.

(d) Effect of Convection Velocity

In addition to the aforementioned factors, the external convection velocity significantly impacts the sub-storehouse’s heat transfer. Experiments were conducted by varying the external convection velocity (2.5 m/s, 3.5 m/s, and 4.5 m/s) while keeping other parameters constant. The measured heat transfer rates for these velocities over 24 h were 436.90 kJ, 508.92 kJ, and 571.33 kJ, respectively. The relationship between heat transfer rate and convection velocity is depicted in Figure 6d.

Based on the observations from Figure 6a, it can be deduced that the thermal conductivity of the material used for the sub-storehouse body has a minimal impact on its heat transfer performance, despite a substantial 564-fold difference in thermal conductivity among various materials. The heat transfer power of the sub-storehouse constructed from different materials only varies by a factor of 1.08. Consequently, attempting to enhance the sub-storehouse’s heat transfer properties by altering the material’s thermal conductivity is not a feasible approach. Adhering to the principle of cost reduction, polyethylene film, which is more affordable, was decisively chosen as the material for the sub-storehouse body.

The analysis of Figure 6b reveals that the sub-storehouse’s body thickness has a minimal effect on its heat transfer performance. Even with a four-fold increase in thickness from 0.2 mm to 0.8 mm, the actual heat transfer decreases by less than 5%. As such, it is not a viable approach to attempt to influence the body thickness of the sub-storehouse to affect the heat transfer. Then, the confidence is in the sturdiness and cost of the storehouse body. Considering cost, the thickness was set to 0.2 mm in this study. Practically, the thickness of the storehouse has to be determined according to cost, size, structure, and external conditions of the storehouse, which requires a specific case analysis.

The analysis of Figure 6c demonstrates that increasing the heat transfer area of the sub-storehouse from 5.2 m^2^ to 7.2 m^2^ results in a 33.94% increase in the sub-storehouse’s heat transfer rate. This finding indicates that increasing the heat transfer area of the sub-storehouse is a highly effective method for optimizing its heat transfer performance. Therefore, in practical applications, the heat transfer area can be appropriately increased to further enhance the sub-storehouse body’s heat transfer performance.

Figure 6d analysis reveals that when the convective wind speed increases from 2.5 m/s to 4.5 m/s, the sub-storehouse’s heat transfer increases by 30.77%. This finding highlights the significant influence of convective wind speed on the sub-storehouse’s heat transfer. Consequently, adjusting the convective wind speed can be a means to improve the sub-storehouse’s heat transfer. Moreover, since the sub-storehouse’s characteristic is to transfer heat without transferring mass, there is no need to be concerned about humidity being affected by the convective wind speed. The arrangement of micro-fans inside the sub-storehouse can be adopted to boost the convective wind speed. However, this method is not as cost-effective as increasing the sub-storehouse’s heat transfer area, as increasing the heat transfer area is a one-time investment.

In the area of heat transfer, Scheffler [40] showed that polyethylene film demonstrates durability that is either on par with or exceeds that of metallic materials, a conclusion that matches our study’s findings. Ni [41] explored aluminum, various commercially important plastics, and thermosetting polymers, indicating that polyethylene/aluminum connections exhibit the highest interfacial conductance. Typically, within a certain range, there is a direct relationship between thermal conductivity and heat transfer efficiency, further supporting the excellent heat transfer characteristics of polyethylene film, which aligns with our observations. Niu [42] investigated the influence of channel wall thickness on heat and moisture transfer, showing that an increase in wall thickness corresponds to a decrease in optimal rotational speed. This result differs from ours and could be due to variations in material attributes. Regarding film thickness, He [43] studied the connection between polyethylene film thickness and planar thermal conductivity, proposing the potential for reducing film thickness to the nanometer level. It is evident that the thickness of polyethylene has little influence on heat transfer, thus reinforcing the reliability of our research outcomes. In terms of heat transfer area, Soylemez [44] established that the dimensions or surface area of the heat exchanger are directly proportional to heat recovery efficiency, a result that aligns with our findings. Concerning convective wind speed, Ding [45,46,47] found through various studies that there is a substantial connection between wind speed and heat transfer, which closely matches our conclusions. Jaydeep [48] further corroborated this by analyzing temperature and velocity distributions in apple cold storage zones through simulations, demonstrating that increased wind speeds result in reduced temperatures within the cold storage and improved temperature uniformity. Overall, many studies have explored the application of phase change materials for achieving energy savings and maintaining stable temperatures [10,11,12,13,14,15]. However, our approach diverges from this conventional path. Our sub-storehouse materials prioritize non-thermal insulation characteristics, focusing on improving heat transfer performance, thereby establishing a unique direction in our research.

### 3.2. Simulation of Temperature Field in Phase Temperature Storage and Optimization of Sub-Storehouse Structure

#### 3.2.1. Analysis of Flow Field and Temperature Field Simulation Results

**Figure 8:**
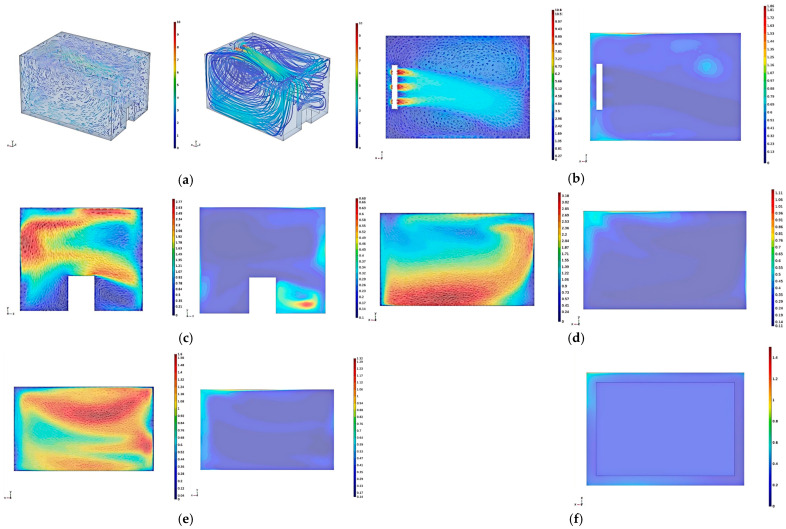
Simulation Results of the Flow Field and Temperature Field in the phase-temperature storage. (**a**) Velocity Vector Diagram and Streamline Diagram of the sub-storehouse Jacket Layer. (**b**) Velocity and Temperature Distribution on the Horizontal Section at the Evaporator Outlet. (**c**) Velocity and Temperature Distribution on the Jacket Layer Section on the Door Side. (**d**) Velocity and Temperature Distribution on the Jacket Layer Section on the Right Side. (**e**) Velocity and Temperature Distribution on the Jacket Layer Section on the Left Side. (**f**) Temperature Distribution on the Horizontal Section 4 m above the Ground.

Figure 8a reveals that the cold air blown out from the evaporator shifts to one side as it moves through the jacket layer above the sub-storehouse and main storehouse, leading to an uneven distribution of the return airflow from both sides. Two vortices are generated in the upper part of the jacket layer, located on either side of the main airflow. These vortices occur because a portion of the airflow does not move downward along the jacket layer on the door side but instead directly returns above the jacket layer to the evaporator’s return air inlet and then flows back from both sides. In particular, it reduces the airflow below the jacket layer on the doorside, where the airflow is blocked by the door while moving downward and flows out to the sides.

Figure 8b (horizontal section at the evaporator outlet) demonstrates that the airflow blown out from the evaporator deviates after traveling a certain distance, resulting in uneven airflow distribution on both sides of the evaporator. When it hits the wall, part of the airflow simply returns along the wall, creating localized vorticies on either side of the main flow. Air movement in these areas of vortex happens at a lower speed of airflow, and the heat cannot dissipate quickly from here, which is why it is important to keep temperatures high in these regions. Additionally, the temperature at the evaporator’s return air inlet is higher as the flow of air passing through it absorbs heat from the external environment, causing the temperature of the returning airflow to rise. The temperature at the cold storage corners near the fan and the walls is also elevated because the airflow deviates from the wall as it returns to the fan inlet. Some of the airflow returns to the return air inlet, while some follows the airflow from the outlet and exits again. This leads to reduced airflow in the corners and along the walls of the cold storage, which results in poor heat exchange performance. Therefore, when designing the internal structure of the sub-storehouse, we need to reduce the recirculation, improve the heat exchange efficiency, and avoid the dead zones of airflow as much as possible.

From Figure 8c (center section of the jacket layer on the door side), it can be observed that a portion of the airflow, blocked by the wall, collides with the door during its downward movement and then flows to the sides of the door. When the airflow is obstructed by the side wall, the airflow reverses and forms a vortex at the corner beneath the door, therefore accumulating heat and elevating temperature, further impairing the heat exchange performance. Consequently, the airflow on this section is asymmetrical.

Figure 8d (center section of the jacket layer on the right side) reveals that the airflow generally follows an arc-like path and recirculates, with a significant difference in flow speed. The majority of the airflow is deflected by the wall and flows toward the bottom surface, then flows across the bottom surface back to the jacket layer on the fan side, with a maximum flow speed of 3.18 m/s. Since a small amount of flow recirculates before reaching the wall, most of the recirculation is found in the upper part of the section. Moreover, the temperature in the upper left area of the section is high in the area around the fan. As a result, the airflow that is blown out from the evaporator is quickly returned towards the fan and does not reach the wall. Therefore, heat is not dissipated from the wall in time. Additionally, along with a high-temperature consequence, this area is also the end of a recirculation zone. Thus, it receives a lot of heat from returned airflow.

Figure 8e (center section of the jacket layer on the left side) shows that most of the airflow recirculates through the upper part of the section, with the maximum recirculation speed reaching 1.6 m/s. Only a small amount of airflow recirculates from the lower part of the section, with a low flow speed insufficient to conduct the heat from the left wall, resulting in higher temperatures on the left side for the same reason as previously explained.

At the center of the jacket layer on the right side, the velocity is 1.79 m/s and the average temperature is 0.19 °C, while on the left side, the velocity is 1.04 m/s and the average temperature is 0.26 °C. The difference between the two is significant, primarily due to the uneven airflow distribution when the evaporator’s air passes through the left and right jacket layers. This issue can be addressed by optimizing the internal structure of the sub-storehouse and modifying the airflow paths.

Figure 8f illustrates that the temperature distribution in the sub-storehouse on the horizontal Section 4 m above the ground is relatively uniform.

#### 3.2.2. Optimization of the Sub-Storehouse Structure in Phase Temperature Storage

**Figure 9:**
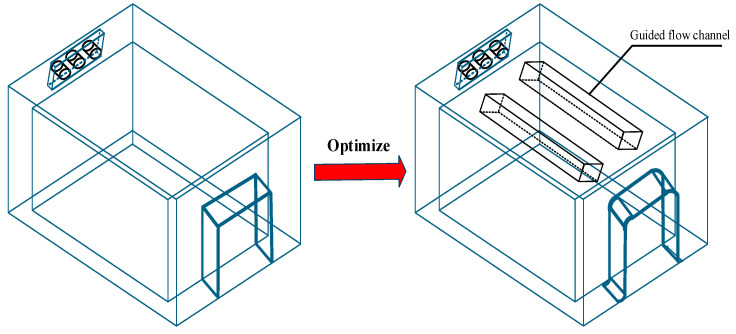
Optimized Model of the Sub-storehouse.

As the simulation results mentioned above reveal issues such as uneven airflow and numerous vortices in the phase temperature storage system, this section focuses on optimizing the structure of the phase-temperature storage. To address the issue of airflow displacement above the jacket layer, a guided flow channel is installed. The dimensions of the guided flow channel are 5 m × 1 m × 1 m (length × width × height), with a distance of 2.2 m from the air outlet and 2 from the side wall. To solve the problem of excessive vortices on the door side, the sharp corners of the door are replaced with rounded edges. The radius of the upper rounded corner is 0.2 m, while the radius of the lower rounded corner is 0.3 m. The modified structure is shown in Figure 9.

**Figure 10:**
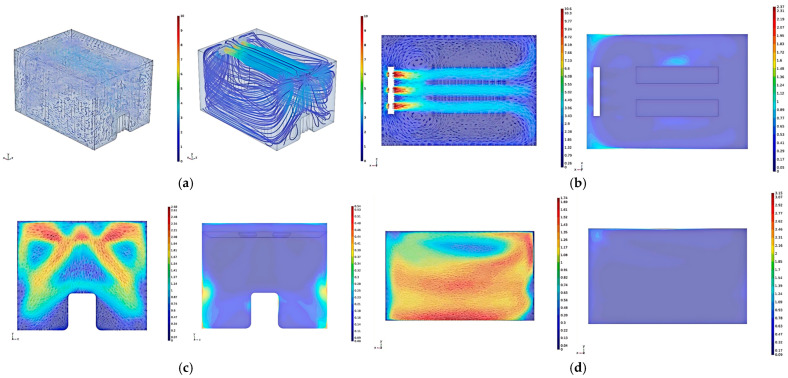
Simulation Results of Flow Field and Temperature Field After Optimization in Phase-temperature Storage. (**a**) Velocity vector diagram and flowline diagram of the corrugated surface jacket layer of the sub-storehouse. (**b**) Velocity and temperature distribution on the horizontal section of the evaporator outlet. (**c**) Velocity and temperature distribution on the jacket layer section on the door side. (**d**) Velocity and temperature distribution on the jacket layer section on the right side.

Figure 10a illustrates that upon exiting the evaporator, the airflow is directed into a guided flow channel. This channel prevents the displacement of airflow above the jacket layer. Subsequently, the airflow is distributed more uniformly to both sides and downward, effectively eliminating recirculation-induced vortices. The flowline distribution within the sidewall jacket layer exhibits enhanced uniformity, indicating an even distribution of airflow without recirculation in the upper and lower regions. The overall flow field is characterized by symmetry and regularity in airflow organization. Consequently, the analysis will focus on representative cross-sections of the jacket layer.

In Figure 10b, the airflow, upon collision with the wall, flows back along the surface, facilitating prompt removal of external heat. However, a vortex is observed near the evaporator outlet. The reason for this phenomenon is related to high-velocity wind at the outlet that generates a localized low-pressure zone. If there is this low-pressure area, it imposes some recirculating airflow, and then the outgoing airflow perturbs that, resulting in the formation of the vortex. The vortex is very small and close to the outlet, but heat discharge is maintained so that there is very little temperature rise in the neighborhood. Nevertheless, elevated temperatures persist in the corners adjacent to the evaporator. A portion of the airflow needed to extend to these corners recirculates back into the evaporator, reducing airflow and, thus, heat transfer efficiency in these areas.

Figure 10c demonstrates that rounding the door corners facilitates improved airflow above the door towards both sides, enhancing the cooling effect below. Although vortices are still present beneath the door, they do not induce localized temperature increases, indicating effective heat removal in the vortex area. However, higher temperatures are observed near the lower side wall of the section. This is attributed to the majority of the airflow, post-wall collision, flowing into the side jacket layer and recirculating. A small fraction of the airflow propagates in the vertical direction along the wall with local vortices and affects heat exchange. The flowline patterns in the figure reveal symmetric distribution of airflow in the door-side jacket layer. This symmetry indicates balanced recirculating airflow on both sides of the sub-storehouse, prompting a focused analysis of airflow velocity and temperature distribution in the center of the right-side jacket layer.

In Figure 10d, the recirculating airflow exhibits a more uniform distribution across the section, with no significant displacement. This represents a substantial improvement in airflow distribution compared to the pre-optimization state. The airflow closely adheres to both side walls, with the mainstream area approximating a rectangular shape. This indicates more evenly distributed airflow across the height of the jacket layer, thus improving the previously uneven flow field distribution across the height of the jacket layer. This uniformity in the flow field correspondingly leads to a more homogeneous temperature distribution within the section. Nevertheless, the same factors persist there as before, creating a localized vortex on the upper left with higher temperatures.

The simulation results of the temperature field and flow field within the optimized sub-storehouse exhibit significant differences compared to those reported by Zargar [49] for a three-layer multi-door large cold storage. These discrepancies can be attributed to variations in structural design, material properties, and the installation location of the experimental sub-library. Despite these differences, the findings align well with Chen’s [50] simulation results regarding the air temperature field in vaccine storage facilities and Mahdi’s [36] results on temperature and flow fields in prototype cold storage units. Consequently, optimizing the sub-storehouse through the integration of guided flow channels and enhancement of door curvature angles markedly improves heat transfer efficiency, thereby enabling more precise and stable temperature control within the sub-storehouse. This approach provides valuable and effective strategies for advancing phase-temperature storage technology.

### 3.3. Analysis of Defrosting Experiment Results

#### 3.3.1. Comparison of Defrosting Energy Consumption

The experimental measurements of total energy consumption for the two defrosting units were recorded as 5.72 kWh and 154.02 kWh, respectively. These data lead to the following conclusion. The total defrosting energy consumption of the phase-temperature storage system is less than 4% of that observed in conventional cold storage. Consequently, the phase-temperature storage system demonstrates an energy efficiency improvement of over 96% compared to conventional cold storage systems.

#### 3.3.2. Study of Defrosting Waste Heat

Table 2 presents data collected over a 168 h (7-day) period, during which the defrosting system of the conventional cold storage operated for a total of 572 min. This operation resulted in an electricity consumption of 36.359 kWh and a melt-water collection of 20.588 kg. Applying Formula (12), the energy expended for frost melting was calculated to be 7662.737 units. When converted to electrical units, this equates to 2.128 units, indicating that the actual energy consumption for defrosting is merely 2.128 kWh. The remaining energy, approximately 34.231 kWh, is dissipated as defrosting waste heat into the cold storage area, representing a substantial loss of 94.13%.

From the aforementioned results, it is evident that phase-temperature storage with the same volume achieves a 70.59% reduction in energy consumption compared to conventional cold storage for defrosting units, both in terms of energy consumption and defrosting waste heat recovery. Furthermore, Dong [51] experimentally investigated the heat supply and energy consumption during reverse cycle defrosting operations of air-source heat pumps, revealing that defrosting efficiency can reach up to 60.1%. Qu [52] further demonstrated that the reverse cycle defrosting method based on Thermal Energy Storage (TES) significantly reduces both defrosting time and energy consumption in cascade air-source heat pumps. Ye [53] conducted a comparative analysis of the performance of reverse circulation defrosting (RCD) and hot gas bypass defrosting (HGBD) in air-source TCHP water heaters, concluding that the RCD method not only achieves lower power consumption but also shorter defrosting times. The HGBD method has a negligible impact on the system’s heat capacity. Jang [54] investigated optimal control strategies for hot gas and proposed a continuous heating approach that eliminates the need for additional equipment in high-capacity cycles of 30 kW or higher, effectively addressing the issue of air-source heat pumps interrupting heat supply during defrosting. Melo [55] conducted an experimental evaluation of a domestic refrigerator defrosting system, achieving a maximum efficiency of approximately 48% when using a glass tube heater in a power ladder configuration. Despite these studies emphasizing advantages, they all involve cyclical processes of frost formation and subsequent defrosting, which inherently lead to energy losses. In contrast, the phase temperature storage reservoir can directly and efficiently prevent evaporator frosting through its unique coupling structure. Frosting is only minimally observed during the initial preservation phase, primarily due to residual moisture in the air within the main storehouse or airflow between the main storehouse and sub-storehouse during pre-cooling. This characteristic makes the phase-temperature storage reservoir highly energy-efficient and worthy of further promotion.

### 3.4. Results of Simulation Analysis of Goods Stored in Sub-Storehouse

**Figure 11:**
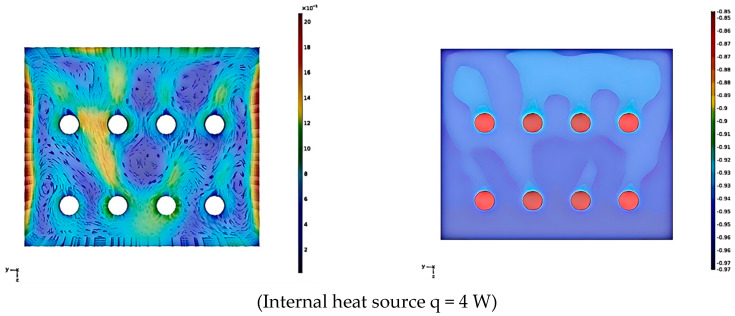

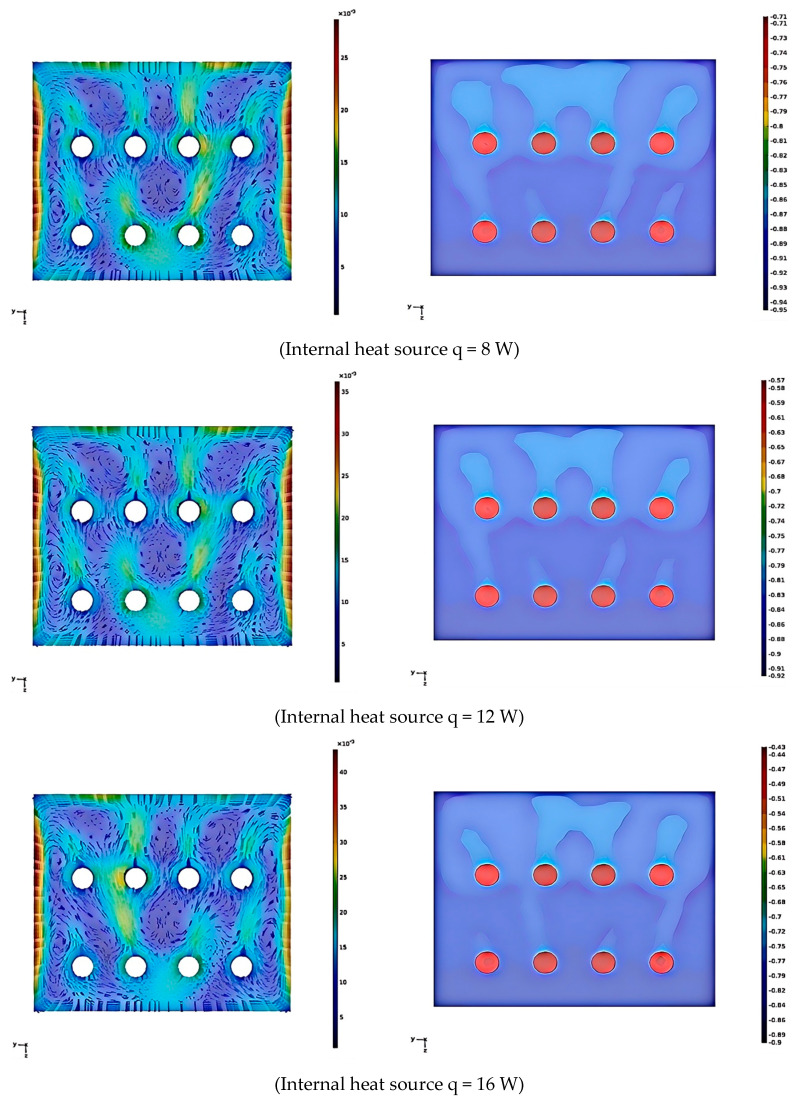
Velocity and temperature distribution of the middle longitudinal section of the sub-storehouse under different q.

The simulation diagram of the velocity and temperature distribution of the middle longitudinal section of the sub-storehouse under different q conditions is shown in Figure 11.

As illustrated in Figure 11, despite variations in internal heat sources within the cargo, the overall distribution trends of the velocity field and temperature field on the cross-section remain consistent. The air surrounding the cargo experiences an increase in temperature and a decrease in density due to the influence of internal heat sources, thereby inducing upward natural convection. Owing to the relatively low temperature of the walls, the density of the airflow increases upon contact with the wall. Upon encountering the wall, the high-temperature airflow descends while simultaneously transferring heat to the wall. This process establishes the upper and lower circulation of the airflow, enhancing convective heat transfer between the cargo and the wall. Although the upward airflow near the cargo interacts with the downward airflow near the wall, forming a vortex between them, the temperature distribution reveals that the temperature at the vortex does not rise significantly. This indicates that the vortex generated by natural convection has minimal impact on heat transfer, resulting in a relatively uniform temperature distribution within the sub-storehouse. With the increase in internal heat sources, the cargo temperature rises markedly, leading to a greater temperature imbalance within the reservoir and an increase in flow rate. Specifically, when the internal heat source reaches 16 W, the maximum flow rate near the wall is 0.04 m/s, while the gas flow rate around the cargo ranges between 0.015 and 0.025 m/s. Despite this, the overall flow rate remains relatively small, minimizing the dry consumption of cargo. Consequently, even when the sub-reservoir contains stored goods, the temperature distribution remains highly uniform, and the overall airflow velocity is low, ensuring high-quality storage conditions.

Leo [56] demonstrated that a tray height of 0.3 m exhibited superior temperature performance compared to heights of 0.0 m, 0.6 m, and 0.9 m, which is consistent with the findings of this study. Liu [18] further deduced through computational fluid dynamics (CFD) modeling that slower airflow at the downstream end led to elevated temperatures and the formation of localized vortices, thereby supporting the results of this study. Jaydeep [48] conducted simulations of temperature and flow velocity within an apple refrigeration environment, revealing that apples in the upper layer maintained slightly higher temperatures than those in the lower layer, which indirectly verifies the reliability of the results presented in this study.

### 3.5. Comparison of Preservation Effects Between Phase-Temperature Storage and Conventional Cold Storage

#### 3.5.1. Impact of Different Storage Environments on Apple Respiration Rate

**Figure 12:**
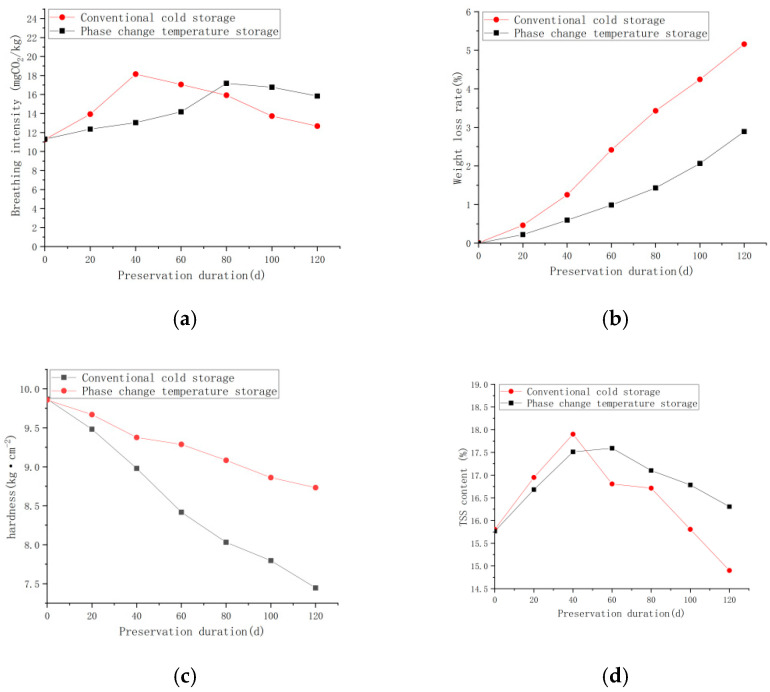
Analysis of Apple Preservation Effect in Different Cold Storage Environments. (**a**) The effect of different cold storage methods on apple respiration rate. (**b**) The effect of different cold storage methods on apple weight loss rate. (**c**) The effect of different cold storage systems on apple firmness. (**d**) The effect of different cold storage systems on apple TSS (Total Soluble Solids) content.

Apples, being climacteric fruits, exhibited a significant peak in respiration rate during storage in both treatment groups, as illustrated in Figure 12a. However, the relationship between respiration rate and storage environment is multifaceted. From day 0 to day 40, a gradual increase in respiration intensity was observed in apples from both groups. The conventional cold storage group reached its respiration peak around day 40, whereas the phase-temperature storage (PTS) group attained its peak approximately on day 80. The maximum respiration rates recorded for the two groups were 18.15 CO_2_mg·kg^−1^·h^−1^ and 17.05 CO_2_mg·kg^−1^·h^−1^, respectively. Throughout the entire storage period, the respiration rate in the conventional cold storage group demonstrated a rapid increase in the initial stages, followed by a peak and a subsequent swift decline. Conversely, the PTS group maintained a comparatively low respiration rate throughout the storage duration, with a lower peak respiration rate than that observed in the conventional cold storage group. This disparity can be attributed to the reduced temperature fluctuations in the PTS environment. Since respiration rate is indicative of post-harvest ripening, aging, and internal nutrient consumption in fruits, the PTS storage method proves more effective in decelerating the aging process of fruits and vegetables.

#### 3.5.2. Impact of Different Storage Environments on Apple Weight Loss Rate

It is shown in the present research that a weight loss rate exceeding 5% results in a decrease in fruit quality and quality attributes. These effects include wrinkling and a lackluster appearance of the fruit skin, loss of crispness in the flesh, and reduction of the economic value of the apple. Harvested apples are active organic entities that continue to respire post-harvest. Weight loss is inevitable because the flow of water and nutrients that are supplied by the tree has ceased [57]. As illustrated in Figure 12b, a consistent increase in weight loss rate was observed across all storage environments over time. However, the rate of weight loss varied significantly among different storage conditions. For instance, after a 120-day storage period, the weight loss rates for the PTS and conventional cold storage groups were recorded at 2.89% and 5.16%, respectively. These data clearly indicate that the PTS environment resulted in a substantially lower weight loss rate compared to conventional cold storage methods. Consequently, the PTS storage method can be considered the most efficacious in mitigating fruit weight loss.

#### 3.5.3. Impact of Different Storage Environments on Apple Firmness

Changes in cell wall material composition caused by fruit ripening, as well as aging processes, are studied. These changes result in reduced fruit firmness and, thus, fruit softening, causing a quality and market value compromise in the fresh produce resulting from these changes. In Figure 12c, apples in both experimental groups were found to have declined in firmness during storage at different rates. For instance, the firmness was measured at day 60 of storage as 9.29 kg·cm^−2^ for the PTS group and 8.42 kg·cm^−2^ for the conventional cold storage group. In both groups, firmness then continued to decrease, but the decrease in the conventional cold storage group was more rapid. At the end of the storage period, the firmness of apples in the PTS group decreased by 11.41%, while the firmness of apples in the conventional cold storage group decreased by 24.54%. By achieving a reduction in temperature fluctuations, as seen in PTS storage, these findings show that controlling temperature fluctuations is more useful for maintaining apple firmness and overall quality during storage.

#### 3.5.4. Impact of Different Storage Environments on Apple Total Soluble Solids (TSS) Content

TSS content serves as a crucial indicator of fruit quality [58]. As illustrated in Figure 12d, both experimental groups exhibited a trend of gradual increase, followed by a decrease in TSS content over the entire storage duration. This pattern may be attributed to the fact that the apples used in the experiment were not fully mature at the time of harvest. Consequently, their maturity continued to progress during the early stages of storage, resulting in an initial increase in TSS content. As the apples started becoming mature and aging, various physiological activities consumed internal nutrients in the later stages of storage, meaning the TSS content tended to decrease. During the increasing phase, the conventional cold storage group reached its peak TSS value of 17.9% around day 40, while the PTS group attained its peak value approximately on day 60, notably later than the conventional cold storage group. At the conclusion of the storage period, the TSS contents in the PTS and conventional cold storage groups were recorded at 16.3% and 14.9%, respectively. These results indicate that the PTS environment, characterized by more precise temperature control and reduced temperature fluctuations, is superior in maintaining TSS levels.

Through the comparative analysis of apple storage experiments, phase-temperature storage has been shown to substantially suppress the physiological metabolism of apples and effectively retard their aging process. In contrast to conventional cold storage, apples stored under phase-temperature storage conditions display significantly lower respiratory rates, a more gradual decline in hardness, reduced weight loss, and better quality retention. Consequently, phase-temperature storage demonstrates clear advantages over conventional cold storage in maintaining apple quality during post-harvest storage and extending shelf life.

## 4. Conclusions

This study encompassed a comprehensive investigation of the PTS system. Initially, materials for the sub-storehouse were meticulously selected. Subsequently, through advanced simulation techniques, a more optimal structure for the PTS was established and refined. The energy-saving efficacy of the PTS defrosting process was validated, and comparative apple preservation experiments were conducted to juxtapose the storage effects of PTS and conventional cold storage systems. The salient findings are delineated as follows:

1. Experimental results demonstrated that the thermal conductivity of the sub-storehouse material exhibits a strong correlation with the storage area and airflow velocity. Conversely, the thickness and type of material were found to exert a relatively minor influence on thermal performance;
2. The top jacket layer of the sub-storehouse was equipped with an innovative guided flow channel. Additionally, the storage door geometry was modified from an angular to a curved configuration, a marked improvement in airflow distribution uniformity within the jacket layer. Vortex formation at the door was significantly mitigated, and the internal temperature of the sub-storehouse decreased by 0.14 °C. These results provide compelling evidence of enhanced heat transfer performance in the optimized PTS structure;
3. Under the same external conditions, an energy savings of over 96% for the PTS compared to conventional cold storage. Besides this, the PTS, owing to its unique structural design, achieved a reduction in overall energy consumption of approximately 70.5 kwh when considering both defrosting system operation and heat recovery;
4. Software is used to simulate the heat transfer of the walls and goods by convection as the intensity of the internal heat source increases and the cargo temperature rises, resulting in greater temperature non-uniformity and enhanced flow velocities. Despite these variations, the overall flow velocity remains relatively low, and the temperature distribution remains largely uniform, thereby maintaining optimal storage conditions for goods;
5. Apples stored in the PTS exhibit superior quality characteristics compared to those in conventional cold storage. Specifically, PTS-stored apples demonstrated higher firmness and TSS content, coupled with lower weight loss rates and respiration intensity.

It is evident that phase-temperature storage represents an innovative approach to fruit and vegetable preservation, integrating the advantages of conventional cold storage systems. By employing a distinctive main sub-coupling temperature-control technology in conjunction with heat and mass transfer mechanisms, this system achieves frost-free operation in the evaporator while maintaining high humidity levels within the sub-storehouse. This enables precise regulation of both temperature and relative humidity, effectively reducing the respiration intensity of fruits and vegetables during storage. As a result, it prolongs the freshness period and ensures the quality of stored produce. Although some practical application cases and experiences require further refinement, the primary research focus will be on leveraging phase-temperature preservation technology and equipment to develop accurate, energy-efficient, and low-residue storage solutions for fruits and vegetables.

## Figures and Tables

**Table 1:** Ice temperature values of common fruits and vegetables.

Name	Apple	Cucumber	Kiwi Fruit	Orange	Banana	Onion	Eggplant
Freezing Point (°C)	~3–3.8	~2.5–2.7	~3.3–3.7	~1.5–1.6	2~.2–2.7	~1.8–2.0	~2.2–2.8

**Table 2:** Conventional cold storage frost energy consumption and frosting amount.

Cold Storage Operation Time (h)	Defrosting Unit Energy Consumption (kWh)	Melting Water Volume (kg)	Melting Heat Absorption Q (kJ)	Melting Heat Absorption Energy Consumption (kWh)	Defrosting Waste Heat (kWh)	Defrosting Energy Consumption Utilization Rate
0	0	0	0	0	0	
12	2.448	1.426	530.743	0.149	2.302	6.09%
24	5.264	2.907	1081.986	0.299	4.964	5.68%
36	7.956	4.419	1644.618	0.457	7.499	5.74%
48	10.649	5.876	2186.750	0.608	10.041	5.71%
60	13.219	7.399	2753.937	0.765	12.455	5.79%
72	15.425	8.899	3312.014	0.921	14.503	5.97%
84	17.871	10.349	3851.869	1.071	16.800	5.99%
96	20.319	11.812	4396.278	1.222	19.097	6.01%
108	22.277	13.232	4924.742	1.369	20.908	6.15%
120	24.603	14.701	5471.429	1.520	23.083	6.18%
132	28.275	16.304	6068.229	1.686	26.590	5.96%
144	30.968	17.608	6553.415	1.820	29.148	5.88%
156	34.028	19.003	7072.768	1.965	32.063	5.77%
168	36.359	20.588	7662.737	2.128	34.231	5.85%

## Data Availability

The original contributions presented in the study are included in the article, and further inquiries can be directed to the corresponding author.
